# Genome-wide analysis of the NAC transcription factor family in broomcorn millet (*Panicum miliaceum* L.) and expression analysis under drought stress

**DOI:** 10.1186/s12864-020-6479-2

**Published:** 2020-01-30

**Authors:** Zhongying Shan, Yanmiao Jiang, Haiquan Li, Jinjie Guo, Ming Dong, Jianan Zhang, Guoqing Liu

**Affiliations:** 1 Institute of Millet Crops, Hebei Academy of Agriculture and Forestry Sciences, Shijiazhuang, 050035 Hebei China; 2 College of Ecology and Garden Architecture, Dezhou University, Dezhou, 253023 China; 3 Key Laboratory of Minor Crops in Hebei, Shijiazhuang, 050035 Hebei China

**Keywords:** Broomcorn millet, *NAC* genes, Gene expression, Drought stress

## Abstract

**Background:**

Broomcorn millet is a drought-tolerant cereal that is widely cultivated in the semiarid regions of Asia, Europe, and other continents; however, the mechanisms underlying its drought-tolerance are poorly understood. The NAM, ATAF1/2, and CUC2 (NAC) transcription factors form a large plant-specific gene family that is involved in the regulation of tissue development and abiotic stress. To date, NAC transcription factors have not been systematically researched in broomcorn millet.

**Results:**

In the present study, a total of 180 *NAC* (*PmNAC*) genes were identified from the broomcorn millet genome and named uniformly according to their chromosomal distribution. Phylogenetic analysis demonstrated that the PmNACs clustered into 12 subgroups, including the broomcorn millet-specific subgroup Pm_NAC. Gene structure and protein motif analyses indicated that closely clustered *PmNAC* genes were relatively conserved within each subgroup, while genome mapping analysis revealed that the *PmNAC* genes were unevenly distributed on broomcorn millet chromosomes. Transcriptome analysis revealed that the *PmNAC* genes differed greatly in expression in various tissues and under different drought stress durations. The expression of 10 selected genes under drought stress was analyzed using quantitative real-time PCR.

**Conclusion:**

In this study, 180 *NAC* genes were identified in broomcorn millet, and their phylogenetic relationships, gene structures, protein motifs, chromosomal distribution, duplication, expression patterns in different tissues, and responses to drought stress were studied. These results will be useful for the further study of the functional characteristics of *PmNAC* genes, particularly with regards to drought resistance.

## Background

Transcription factors (TFs) play an important role in controlling a variety of vital growth and development processes, such as signal transduction, cellular morphogenesis, and response to environmental stressors, during the growth and development of plants [1, 2]. The NAC family is one of the largest groups of plant-specific TFs [3]. The term NAC derives from three proteins: no apical meristem (NAM), Arabidopsis transcription activation factor (ATAF)1/2, and cup-shaped cotyledon (CUC2). Typical NAC proteins exhibit a highly conversed N-terminal DNA-binding domain containing ~ 150 amino acids, which is divided into five subdomains (A–E) [4].

NAC TFs play important regulatory roles in various aspects of plant growth, development, and adaptation to the environment, including in shoot apical meristem formation [5], cell division and expansion [6, 7] nutrient remobilization [8], flower formation [9], lateral root development [10, 11], leaf senescence [12–16], secondary cell wall biosynthesis [3, 17–20], fiber development [19, 21, 22], fruit ripening [23, 24], seed development [25], response to pathogen infection [26–30], and abiotic stress tolerance [31–33]. Many studies have confirmed that many *NAC* genes play crucial roles in the regulation of plant tolerance to drought. Overexpression of *CarNAC4*, a chickpea TF, enhanced drought tolerance in *Arabidopsis thaliana* [34]. *TaNAC29*, a NAC TF from wheat, enhanced salt and drought tolerance in transgenic *A. thaliana* [35]. Overexpression of the *Miscanthus lutarioriparius* NAC gene *MlNAC5* conferred enhanced drought and cold tolerance in *A. thaliana* [36]. *NAC* genes have also been found to increase tolerance to drought stress in rice. For instance, drought resistance in rice was enhanced by the overexpression of *SNAC1* [37], *ONAC022* [38], and *ONAC045* [39]. *OsNAC10*-overexpressing rice plants showed an increased grain yield under both normal and drought conditions [40]. Therefore, NAC family genes are crucial regulators of plant tolerance to drought.

Broomcorn millet (*Panicum miliaceum* L.), also called proso millet, common millet, and hog millet, is a short-season, drought-tolerant, and barren-tolerant cereal that is widely cultivated in the semiarid regions of Asia, Europe, and other continents. The grains of broomcorn millet not only have high nutritional value, containing abundant proteins, starch, and a variety of vitamins and minerals, but also have medicinal value and are used in millet wine and other products. The NAC gene family has been widely studied in many species, such as *A. thaliana* [41], rice [41], wheat [42, 43], tartary buckwheat [44], maize [45, 46], foxtail millet [47], soybean [48], potato [49], Chinese cabbage [50], pepper [51], cassava [52], melon [53], physic nut [54], apple [55], and pigeon pea [56]. However, no systematic study of the *NAC* family in broomcorn millet is available. The genome of broomcorn millet was recently published, providing an important resource for further molecular research in this species [57]. In addition, large RNA sequencing (RNA-seq) expression data from different tissues are also available [58]. Based on these data, *NAC* gene family members in broomcorn millet were identified in the present study. A phylogenetic tree was constructed, gene exon/intron and conversed motif structural analyses were performed, chromosomal location and synteny analysis were carried out, and the tissue-specific expression patterns of *NAC* genes in broomcorn millet were surveyed. In addition, the differential expression of *NAC* genes was analyzed in broomcorn millet under drought conditions using transcriptomics and quantitative real-time PCR (qRT-PCR). This research is the first to detail the *NAC* gene family in broomcorn millet, which may help elucidate the molecular mechanisms underlying drought stress responses in this important food crop.

## Results

### Identification and phylogenetic analysis of the PmNAC genes in broomcorn millet

A total of 180 *PmNAC* genes were identified and were named *PmNAC001* to *PmNAC180* according to their chromosome location. These were confirmed and used for further analyses (Additional file 1: Table S1). All the PmNAC proteins contained a conserved NAC domain (PF01849) or NAM domain (PF02365).

**Fig. 1:**
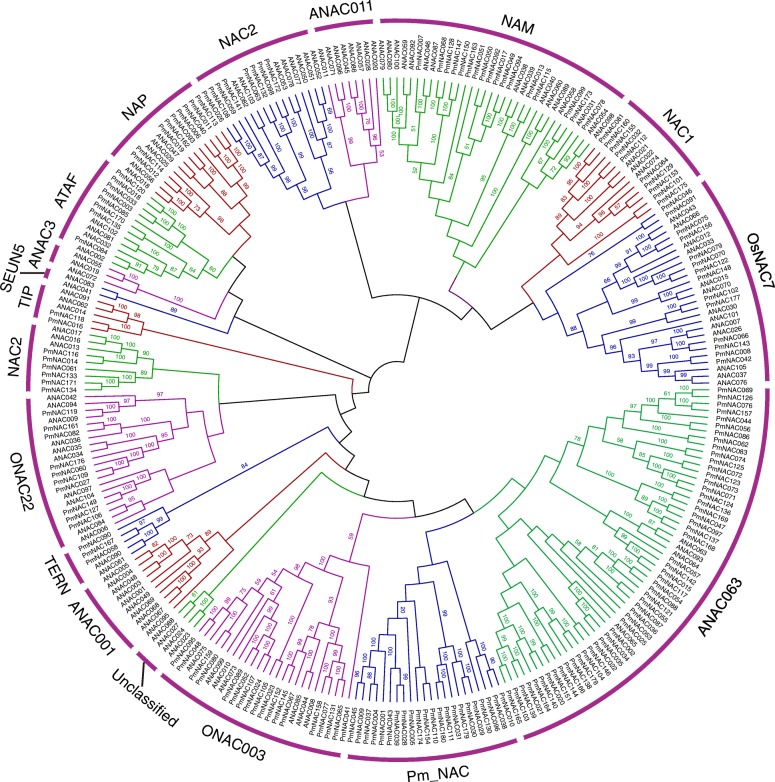
Phylogenetic analysis of NAC proteins of broomcorn millet and *Arabidopsis*. The phylogenetic tree was constructed using the neighbour-joining method in MEGA-X

Phylogenetic analysis of the NAC proteins from broomcorn millet and *Arabidopsis* was conducted to explore the evolutionary relationships among broomcorn millet NAC proteins. The results demonstrated that the PmNAC proteins could be divided into 12 subgroups according to clade and the classification from *Arabidopsis*, including a broomcorn millet-specific subgroup named Pm_NAC (Fig. 1, 2a). In our analysis, no NAC members from the subgroups ANAC001, SEUN5, ANAC3, and ANAC011 were identified. Subgroup ANAC063 contained the most PmNAC proteins, namely 52, while subgroup TIP contained only two PmNAC proteins.

**Fig. 2:**
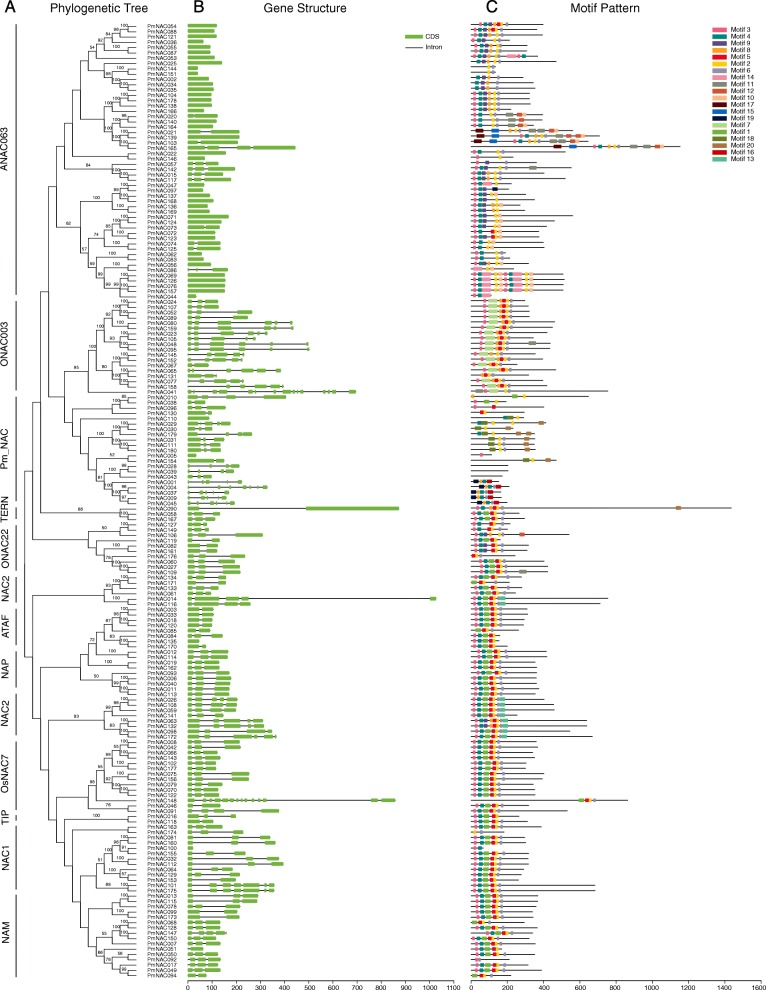
Phylogenetic relationships, gene structure, and architecture of conserved protein motifs in NAC genes from broomcorn millet. **a** A phylogenetic tree was constructed by MEGA-X using the NJ method. **b** Structures of the 180 putative broomcorn millet *NAC* genes. Light green boxes indicate exons and black lines indicate introns. **c** Motif distribution of NAC proteins. Different motifs are indicated by different colours for motifs 1–20. The sequence information for each motif is provided in Additional file 1: Table S3

### Protein properties and sequence analyses

The protein properties were analysed, and the results are summarized in Additional file 1: Table S2. The length and molecular mass of the PmNAC proteins varied greatly, with lengths ranging from 67 to 1435 amino acids (aa) and molecular weights (MWs) ranging from 7.53 to 162.48 kDa. The theoretical isoelectric point (pI) varied greatly from 4.13 to 11.38. Most PmNAC proteins (149 out of 180) were considered unstable due to the instability index being higher than 40. The PmNAC proteins contained a predicted aliphatic index ranging from 45.59 to 101.24. All PmNAC proteins were predicted to be hydrophilic due to the relatively low average hydropathy (GRAVY) value (< 0), with the exception of PmNAC005 and PmNAC165.

Gene structural diversity is an important component of gene family evolution and further supports phylogenetic groupings [59]. Gene structure analysis indicated that the number of introns of *PmNAC* genes varied from 0 to 14 (Fig. 2b), with 44 *PmNAC* genes lacking introns. Most *PmNAC* genes (134 out of 180) contained one to six introns. *PmNAC41* had the highest number of introns (14), followed by *PmNAC148* with 13 introns. Generally, closely clustered *PmNAC* genes in the phylogenetic tree exhibited similar exon-intron structures. Most *PmNAC* genes had no introns in subgroup ANAC063, and there was an average of four introns in subgroup ONAC003.

To further investigate the structural features of broomcorn millet NACs, the conserved motif distributions were analysed. A total of 20 conserved motifs (referred to as motifs 1–20) were identified by MEME, with more motifs located within the N-terminal region than within the C-terminal region (Fig. 2c). The features of these protein motifs are listed in Additional file 1: Table S3. In this analysis, most of the closely related members in the phylogenetic tree showed common motifs in terms of alignment and position, which suggested that the NAC members that clustered along the same branch may possess similar biological functions.

### Chromosomal location and synteny analysis of *PmNAC* genes

**Fig. 3:**
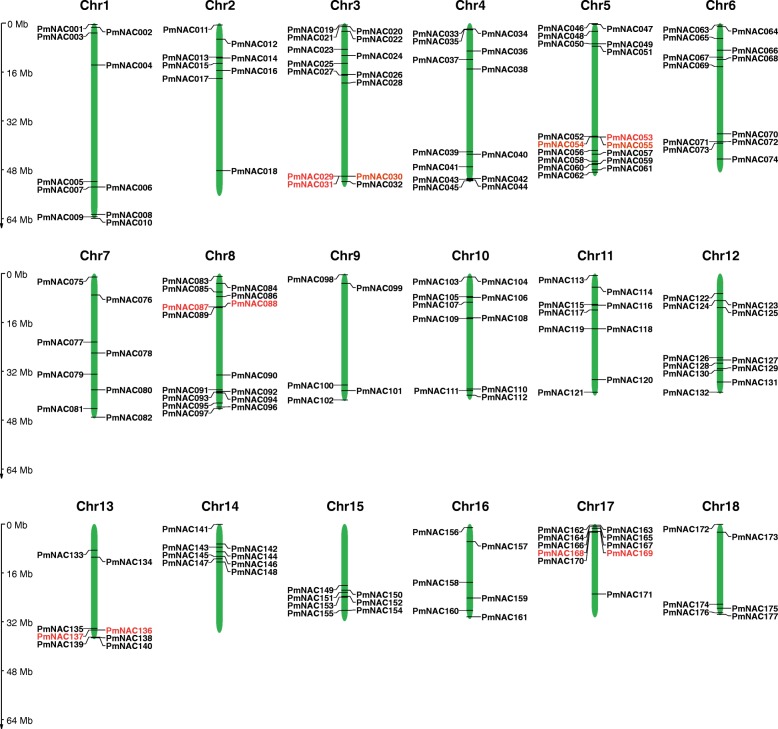
Positions of NAC gene family members on broomcorn millet chromosomes. Tandem duplicated genes are indicated in red

All the *PmNAC* genes were unevenly distributed on broomcorn millet chromosomes, except *PmNAC178–180* (Fig. 3). Chromosome 5 (Chr5) contained the highest number of *PmNAC* genes (*n* = 17), followed by Chr8 (*n* = 15) and then Chr3, which had 14 members. In addition, 13, 12, and 11 *PmNAC* genes were detected on Chr4, Chr6, and Chr12, respectively. Ten *PmNAC* genes were found on each of Chr1, Chr10, and Chr17. Nine genes were located on Chr11 and seven *NAC* genes were located on Chr15. Eight *PmNAC* genes were distributed on each of Chr2, Chr7, Chr13, and Chr14, and six *PmNAC* genes were found on both Chr16 and Chr18. There were only five *PmNAC* genes on Chr9.

**Fig. 4:**
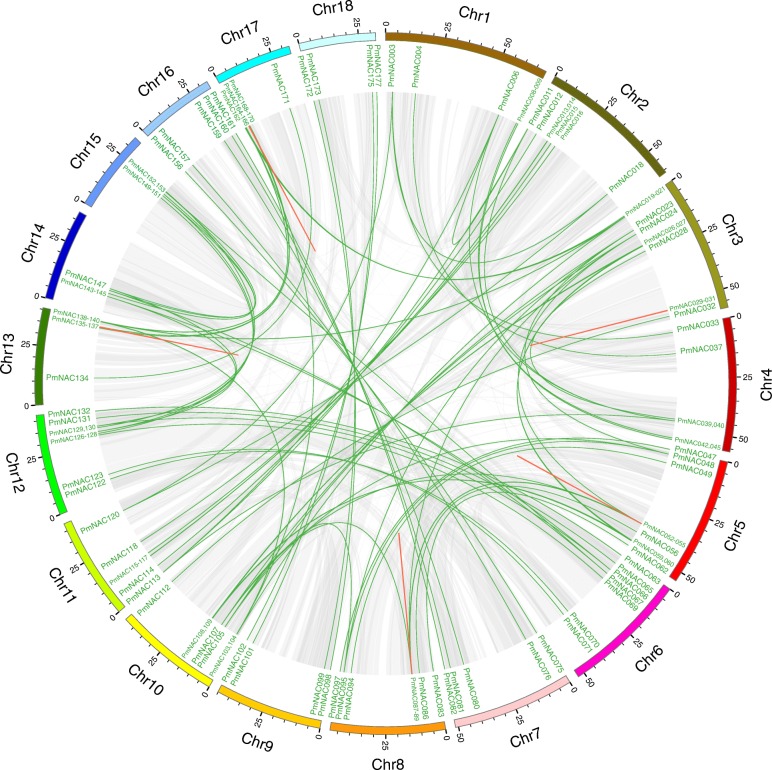
Gene duplication of *PmNAC* genes. Gray lines indicate all synteny blocks in the broomcorn millet genome, the green lines indicate duplicated *NAC* gene pairs, and the red lines indicate tandem duplicated genes

To identify the duplication events in *PmNAC* genes, a collinearity analysis was performed using MCScanX software. There were 84 pairs of segmentally duplicated *PmNAC* genes (Fig. 4) and five groups of tandem duplicated *PmNAC* genes (Figs. 3, 4; *PmNAC029*/*030*/*031*, *PmNAC053*/*054*/*055*, *PmNAC087*/*088*, *PmNAC136*/*137*, *PmNAC168*/*169*). Duplicated genes were the most common on Chr3, followed by Chr5 and Chr10 (Fig. 4).

### Expression pattern analysis of *PmNAC* genes in different tissues

**Fig. 5:**
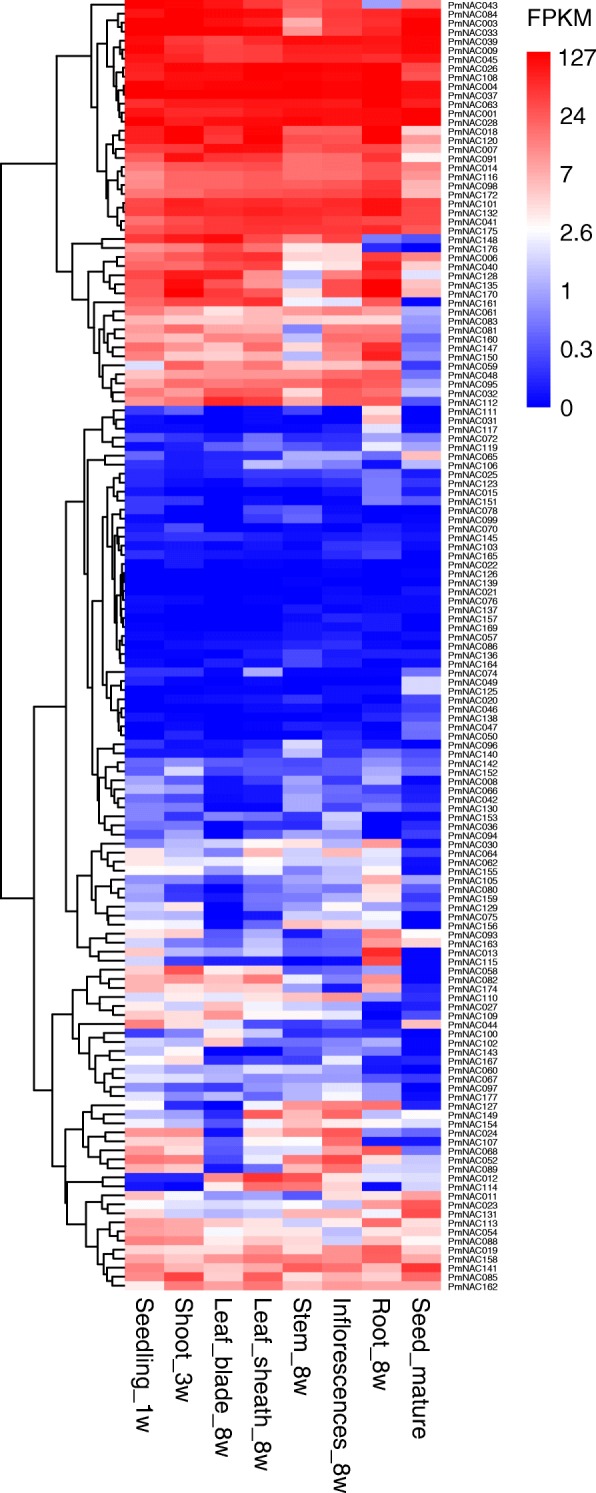
Expression patterns of NAC genes in different tissues. Seedling_1w, Shoot_3w, Leaf_8w, Sheath_8w, Stem_8w, Inflorescences_8w, Root_8w, and Seed_mature indicate tissues from seedlings at one week of age, shoots at three weeks of age, leaf blades, leaf sheaths, stems, inflorescences, and roots at eight weeks of age, and seeds at 12 weeks of age, respectively. FPKM values were used to generate the heatmap with hierarchical clustering analysis. The scale represents the relative signal intensity of the FPKM values

To better understand the function of *PmNAC* genes in broomcorn millet, the transcription levels of *PmNAC* genes in different tissues, i.e., seedlings at one week of age, shoots at three weeks of age, leaf blades, leaf sheaths, stems, inflorescences, and roots at the eight weeks of age, and mature seeds at 12 weeks of age, were investigated using publicly available transcriptome datasets [58]. The Fragments Per Kilobase per Million mapped reads (FPKM) values of the *PmNAC* genes are listed in Additional file 1: Table S4, and a hierarchical clustering analysis and heatmap were generated to display the expression patterns of the *PmNAC* genes (Fig. 5).

The expression of three *PmNAC* genes (*PmNAC055*, *178*, and *179*) was not detected in any analysed tissue, possibly due to variations in spatial and temporal expression patterns. The expression of 15 *PmNAC* genes (*PmNAC005*, *010*, *038*, *051*, *071*, *073*, *087*, *092*, *104*, *118*, *124*, *144*, *166*, *171*, and *180*) was detected only in one tissue. Thirteen *PmNAC* genes (*PmNAC001*, *004*, *009*, *026*, *028*, *037*, *039*, *045*, *063*, *101*, *108*, *132*, and *175*) exhibited high expression (FPKM > 20) in all the tested tissues, suggesting key roles of these genes in tissue development. In addition, of all the analyzed tissues, there were 10, 16, 10, 12, 18, 29, 55, and 27 *PmNAC* genes exhibiting the highest expression in seedlings at one week of age, shoots at three weeks of age, and the leaf blades, leaf sheaths, stems, inflorescences, and roots at eight weeks of age, and mature seed at 12 weeks of age, respectively, demonstrating that different *PmNAC* genes may play different roles in the growth and tissue development of broomcorn millet. The high expression of many *PmNAC* genes (31%) in the roots may be one explanation for the observed rapid response of broomcorn millet to drought stress. Understanding the expression patterns of *PmNAC* genes in different tissues can provide a foundation for identifying functional genes in broomcorn millet.

### Responses of *PmNAC* genes to drought treatments

**Fig. 6:**
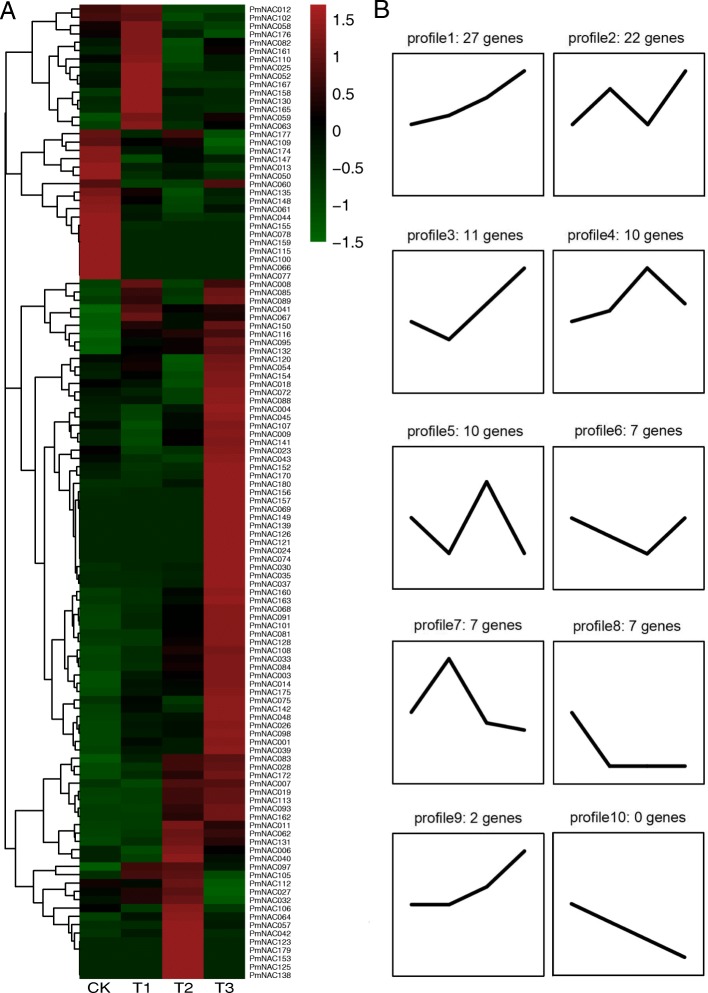
Expression of *PmNAC* genes in response to drought treatment in broomcorn millet. **a** Heatmap showing the relative expression of total *PmNAC* genes at 0 h (CK), 1 h (T1), 3 h (T2), and 6 h (T3) under drought stress. **b** Trend analysis of *PmNAC* gene expression (10 trends)

To detect the dynamic changes in the transcription levels of broomcorn millet *NAC* genes under drought stress, transcriptomic analysis was conducted at 0 h (CK), 1 h (T1), 3 h (T2), and 6 h (T3) under drought treatment (Additional file 1: Table S5). There were 117 *PmNAC* genes detected in the experiment. A heatmap representing the expressions of the detected *PmNAC* genes was constructed (Fig. 6a), and trend analysis was carried out to explore the time-related dynamic changes under drought stress (Fig. 6b). The expression of 27 *PmNAC* genes (*PmNAC003*, *014*, *019*, *026*, *028*, *030*, *033*, *037*, *048*, *068*, *081*, *083*, *084*, *091*, *095*, *098*, *101*, *108*, *113*, *116*, *128*, *132*, *160*, *162*, *163*, *172*, and *175*) increased as the time under drought stress progressed in profile1. The expression of 22 *PmNAC* genes (*PmNAC001*, *008*, *025*, *039*, *041*, *054*, *059*, *063*, *067*, *075*, *082*, *085*, *088*, *089*, *102*, *110*, *120*, *142*, *150*, *154*, *156*, and *161*) showed an ‘N’ shape and peaked at 6 h in profile2, while the expression of 10 *PmNAC* genes (*PmNAC006*, *013*, *040*, *050*, *106*, *109*, *112*, *147*, *174*, and *177*) demonstrated an opposite trend in profile5. The expressions of 11 (*PmNAC004*, *007*, *009*, *023*, *045*, *060*, *093*, *107*, *141*, *152*, and *170*) and seven (*PmNAC018*, *043*, *044*, *061*, *072*, *135*, and *148*) *PmNAC* genes showed similar ‘V’ shape trends in profiles 3 and 6, respectively. The expressions of 10 (*PmNAC011*, *027*, *032*, *042*, *057*, *062*, *064*, *097*, *105*, and *131*) and seven (*PmNAC012*, *052*, *058*, *158*, *165*, *167*, and *176*) *PmNAC* genes first increased but then decreased in profiles 4 and 7, respectively.

**Fig. 7:**
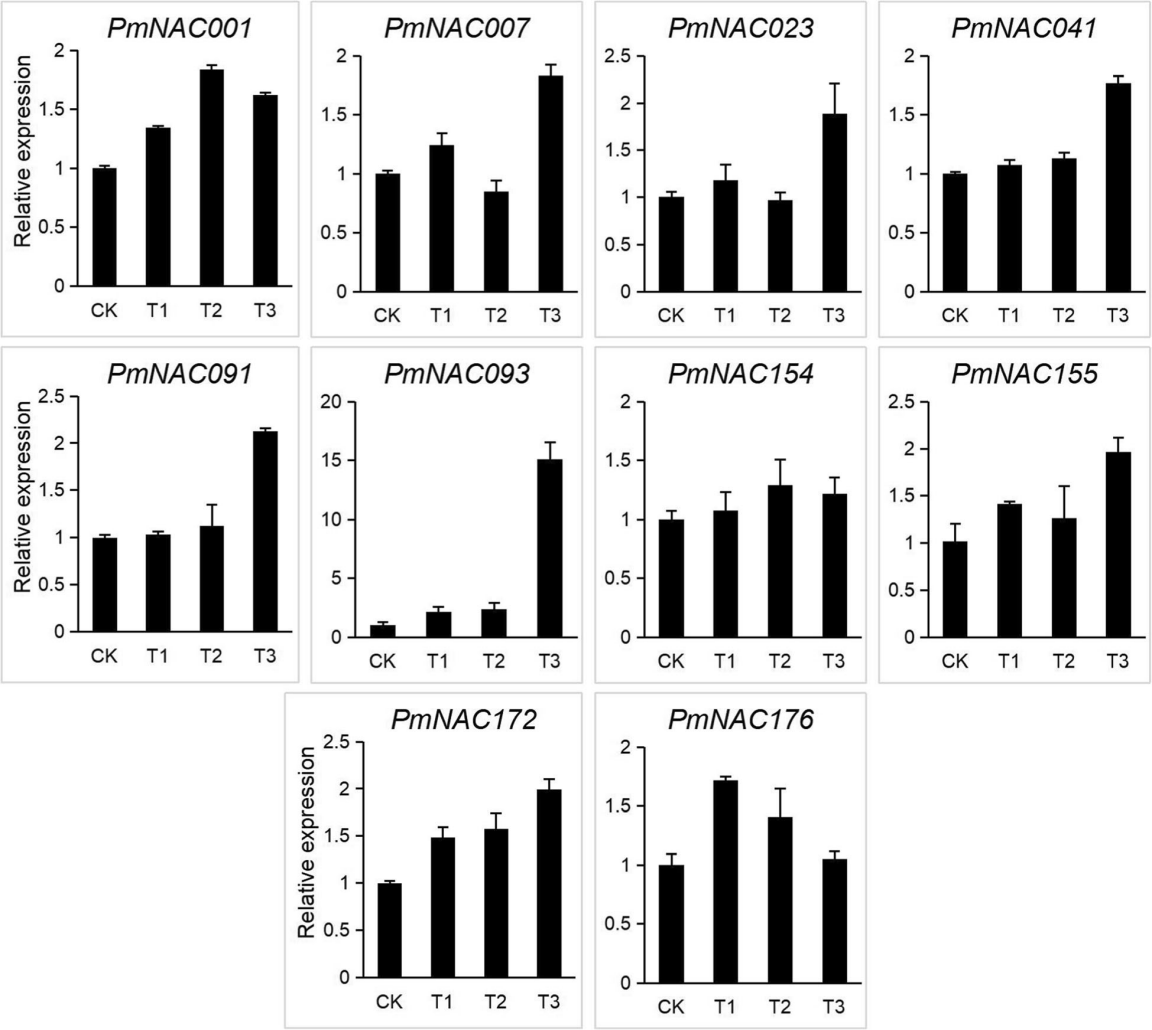
Relative expression of 10 *PmNAC* genes in response to drought treatment using qRT-PCR. CK, T1, T2, and T3 represent the treatments of broomcorn millet exposed to drought for 0 h, 1 h, 3 h, and 6 h, respectively

To verify the RNA-Seq data, 10 *PmNAC* genes (*PmNAC001*, *007*, *023*, *041*, *091*, *093*, *154*, *155*, *172*, and *176*) from different subgroups were randomly selected and tested by qRT-PCR. Most of the tested *PmNAC* genes were up-regulated under drought stress (Fig. 7). Four *PmNAC* genes (*PmNAC041*, *091*, *093*, and *172*) increased with time under drought stress, showing similar trends with the RNA-Seq data. The trend of an initial increase and then decrease in *PmNAC176* was consistent with the RNA-Seq data. The expression level of *PmNAC001* was up-regulated under drought stress compared with the control in both the qRT-PCR results and RNA-Seq data. The expression level of *PmNAC154* also increased under drought stress compared with the control in both methods, except for T2 in the RNA-Seq analysis.

## Discussion

NAC TFs play an important role in regulating plant growth and tolerance against various abiotic and biotic stresses. Most of the NAC TFs studied to date are involved in the regulation of developmental progress and the response to abiotic stress. However, few NAC TFs involved in the drought stress response have been identified in broomcorn millet. Therefore, the goal of this study was to obtain further insight into the expression patterns and putative functions of *PmNAC* genes. In the present study, we investigated the features of *PmNAC* genes at the genome level. Their tissue expression patterns and responses to drought stress were analyzed. Based on gene expression analyses, we identified significant drought-responsive genes. The present study has furthered our understanding of *PmNAC* genes and has provided insight into the functions of *PmNAC* genes.

A total of 180 *NAC* genes were identified in broomcorn millet. The number of *NAC* genes in broomcorn millet was higher than the 117 in *A. thaliana* [41], 151 in rice [60], 104 in pepper [51], 82 in melon [53], 96 in cassava [53], 152 in maize [45], 147 in foxtail millet [47], 152 in soybean [48], and 110 in potato [49], and lower than that of 288 in bread wheat [42] and 188 in Chinese cabbage [50]. There were extensive variations in gene length, predicted protein MW, and protein pI, whereas the gene structures and protein motifs were relatively conserved in the clades, which provided a valuable reference for their analysis and function. This result confirms that genes originating from the progenitors can evolve gradually and expand. It is well known that gene duplication events are important in the rapid expansion and evolution of gene families. A collinearity analysis of the *PmNAC* genes in this study showed that there were 84 pairs of segmental duplications and five groups of tandem duplications. Tandem gene duplication of *NAC* genes has been observed in many species, including *A. thaliana*, rice, potato, and others.

All 180 PmNAC members were divided into 12 subgroups based on their sequence homology and classification from *Arabidopsis* [41]. Phylogenetic analysis divided tartary buckwheat, cassava and pepper NACs into 15, 16 and 14 subgroups, respectively [44, 45, 51]. These data indicated that NAC proteins showed diversity in different species. The analysis regarding the conserved motifs of the broomcorn millet proteins further corroborates the categorization of the PmNAC family. The motifs of transcription factors are often related to protein interaction, transcriptional activity and DNA binding [44]. The conserved motifs in the N-terminus of the NAC genes have highly conserved DNA-binding abilities (Fig. 2 c), which indicates that these motifs are very important for the function of NAC genes. Although conserved motifs were similar among many PmNAC proteins, a number of differences in chemical-physical characteristics were also detected for PmNAC members. These differences may due to the amino acid discrepancies in the non-conserved regions of PmNAC members, implying that different PmNAC proteins may act different functions in their own microenvironments.

The tissue-specific expression patterns of *PmNAC* genes might enable the combinatorial usage of *PmNAC* genes in the transcriptional regulation of different tissues, whereas ubiquitously expressed *PmNAC* genes might regulate the transcription of a broad set of genes. For example, *PmNAC092*, *PmNAC051,* and *PmNAC005* were only detected in the mature seeds and thus may be useful in regulating processes in mature seeds. This phenomenon was also observed in other plants, such as in tartary buckwheat and cassava [44, 52]. Moreover, several reports have indicated that overexpression of tissue-specifically expressed *NAC* genes can promote the development of particular tissues, as *NAC4* from tomato promoted fruit ripening and carotenoid accumulation [23], cotton *NAC transcription factor 1* was involved in secondary cell wall biosynthesis and modification of fibers [19]. Seven highly expressed *PmNAC* genes (FPKM > 20 in all the tested tissues) originated from subgroup Pm_NAC, suggesting the important roles of the broomcorn millet-specific subgroup Pm_NAC in plant growth and development. Thus, the *PmNAC* genes with tissue-specific expression were speculated to have important regulatory functions in the associated tissues, providing insight into their utilization in improving the growth and development of various tissues.

Gene expression pattern analysis can facilitate the determination of gene function. In order to identify the expression profiles of broomcorn millet *NAC* genes in response to drought stress, we performed transcriptomic analysis of the *PmNAC* genes in broomcorn millet plants subjected to 0 h, 1 h, 3 h, and 6 h of drought. Drought treatment increased the expression of 27 *PmNAC* genes originating from nine subgroups, of which the subgroup NAC2 contained the largest number (seven). The seven *PmNAC* genes were closed linked to *ANAC082*, *103*, *013*, *016,* and *017* in *Arabidopsis,* which are involved in the abiotic stress response [61, 62] and mitochondrial retrograde signaling [63, 64]. Notably, some *PmNAC* genes exhibited differential responses to drought at transcriptional levels, although they showed close phylogenetic relationships. Ten *PmNAC* genes were studied under drought stress using qRT-PCR, and most of the *PmNAC* genes showed similar trends with the RNA-Seq data. In combination, the transcriptional response of *PmNAC* genes to drought stress provides a foundation for further investigations into the mechanisms underlying the strong drought tolerance in broomcorn millet.

## Conclusions

To conclude, 180 *NAC* genes were identified in broomcorn millet in the current study. We investigated the features of the *PmNAC* genes at the genome level, and the tissue expression patterns and responses to drought stress were analysed. Genome-wide expression analysis of *NAC* genes in response to drought provides an opportunity to further understand the mechanisms involved in the strong drought-tolerance of broomcorn millet.

## Methods

### Identification and phylogenetic analysis of *NAC* genes in broomcorn millet

The genome of broomcorn millet (accession number GWHAAEZ00000000) was downloaded from the Genome Warehouse in the BIG Data Center (http://bigd.big.ac.cn/gwh). The NAC family databases of *Arabidopsis* and rice were downloaded from The Arabidopsis Information Resource (TAIR9) (www.arabidopsis.org) and the Rice Genome Annotation Project (http://rice.plantbiology.msu.edu/). The PmNAC genes were obtained using two methods. First, the Hidden Markov Model (HMM) files of the NAC (PF01849) and NAM (PF02365) domains were downloaded from Pfam (http://pfam.xfam.org/) for the identification of NAC proteins (E-value < 0.001). Second, all ANAC and OsNAC proteins were used as queries to search against the broomcorn millet database using default parameters. All the possible PmNACs were confirmed using the Conserved Domains Database (CDD) (http://www.ncbi.nlm.nih.gov/cdd/) and PFAM databases (https://pfam.xfam.org). Only the sequences with full-length NAC or NAM domains were considered as PmNAC proteins and used for further analyses.

MEGA-X (https://www.megasoftware.net/) was used for the evolutionary and phylogenetic analyses. A total of 180 PmNACs and 105 AtNACs were used to generate an unrooted phylogenetic tree using the neighbour-joining method with 1000 bootstrap replications and pairwise detection. According to the classification of AtNACs, all the identified PmNACs were divided into different groups. The gene IDs of the *A. thaliana* NAC members were listed in Additional file 1: Table S6.

### Protein properties and sequence analyses

ProtParam (http://web.expasy.org/protparam/) was used for the prediction of the physical and chemical features of the PmNAC proteins. To ensure the subcellular localization of the identified PmNAC proteins, WoLF PSORT was used to predict the protein sequences (https://wolfpsort.hgc.jp/). TMHMM Server v2.0 (http://www.cbs.dtu.dk/services/TMHMM/) was used for the transmembrane helices in the proteins.

To identify the conserved motifs, the MEME tool (http://meme-suite.org/tools/meme) was used with default parameters, except for the maximum number of motifs, which was set to 15. The gene structure of the broomcorn millet NACs was determined using the Gene Structure Display Server (GSDS) 2.0 (http://gsds.cbi.pku.edu.cn/) program.

### Chromosomal mapping and gene duplication analysis

Every *NAC* gene was matched with the chromosomes of broomcorn millet based on the genome annotations of broomcorn millet. MapGene2Chrome (http://mg2c.iask.in/mg2c_v2.0/) was used to draft the map. MCScanX (default parameters) was used to examine duplicated genes [65].

### Expression patterns of *PmNAC* genes in different tissues

The transcriptome data available online were used to explore the expression patterns of *PmNAC* genes in various tissues and different growth stages of broomcorn millet [58]. Seedlings at one week of age, shoots at three weeks of age, leaf blades, leaf sheaths, stems, inflorescences, and roots at eight weeks of age, and mature seeds at 12 weeks of age were sampled to investigate the expression patterns of *PmNAC* genes. Subsequently, FPKM values were calculated to evaluate the gene expression values and the heatmap (Fig. 5) was generated using pheatmap.

### Broomcorn millet plant preparation and drought treatments

The broomcorn millet cultivar Yanshu5 was chosen as the experimental material due to its strong ability to adapt to drought and its relatively high yield [66]. The seeds were planted in flowerpots and grown in a light incubator under the conditions of a 25 °C 14 h photoperiod, and a 22 °C 10 h dark period. They were subjected to drought stress treatments using 20% polyethylene glycol (PEG) 6000 for 0, 1, 3, and 6 h at the 7-d-old stage. The broomcorn millet seedlings were then collected and immediately frozen in liquid nitrogen until RNA extraction.

### Transcriptome and qRT-PCR analysis

The total RNA of the whole seedlings was extracted with TRIzol (Invitrogen) and resequenced using an Illumina HiSeq 4000 (Majorbio) in accordance with the standard Illumina protocol. RNA was used for the reverse transcription of cDNA using HiScript II Q RT SuperMix for qPCR (+gDNA wiper) (Vazyme, China). Primer 5.0 was used to design the primers (Additional file 1: Table S7). The PCR reaction system contained 0.2 μL of forward primer, 0.2 μL of reverse primer, 1 μL of cDNA, 5 μL of 2 × ChamQ SYBR qPCR Master Mix (Vazyme, China), and 3.6 μL of nuclease-free H_2_O. The protocol was as follows: 95 °C for 30 s, followed by 45 cycles of 95 °C for 10 s, and 60 °C for 30 s. Each reaction was performed three times, and the 2^-ΔΔCT^ method [67] was used to calculate the relative gene expression levels.

## Supplementary information

**Additional file 1: Table S1.** List of the NAC sequences in broomcorn millet. **Table S2.** Protein property of PmNAC proteins. **Table S3.** The structural features of motif 1–20. **Table S4.** Expression (FPKM) of *PmNAC* genes in different tissues. **Table S5.** Expression (FPKM) of *PmNAC* genes in response to drought treatment in broomcorn millet. **Table S6.** The gene ID of NAC members from *Arabidopsis thaliana*. **Table S7.** Sequences of primers used in qRT-PCR.

## Data Availability

All data generated or analyzed during this study are included in this article and its additional files.

## Abbreviations

Chr: Chromosome
FPKM: Fragments Per Kilobase of transcript per Million mapped reads
TFs: Transcription factors
